# Annotations

**Published:** 1904-06-25

**Authors:** 


### Annotations.

### Home Care of the Insane.

At the International Home Relief Congress recently held in Edinburgh, a paper on the Scottish system of boarding out lunatics was read by Dr. Alex. Robertson. The special and outstanding feature of this system is the boarding in houses previously licensed by the Lunacy Board of not more than four harmless insane persons, it is an outcome of the Lunacy Act of 1857. In the first few years after the passing of the Act there was much difficulty in obtaining suitable houses, but whereas the number of lunatics boarded with private persons in 1881 was 610, the figures had increased to 1,683 in 1900. The supervision of the patients and their guardians is carefully organised. The doctor of the parish visits all patients in private houses at least once in three months, and enters in a register kept for the purpose a detailed report of each visit. The local inspector of the poor pays a visit every six months and similarly records the results of his inspection. Delegates from the Council of the parish to which the patients are chargeable make an official visit once a year, or sometimes more often. The Chief Commissioner in his report for 1890 dealing with this system, states that "the contentment and happiness which prevail among the boarded-out are very gratifying, and contrast very strongly with the complaints and the appeals for discharge which are so common among the inmates of asylums." As a question of economy, also, the system has proved a great advantage. In the discussion which followed Dr. Robertson's paper, some of the speakers were anything but enthusiastic about the boarding-out system, one gentleman going so far as to say that the inspectors did not perform their duties properly, and that whilst the recoveries in asylums numbered 45 per cent., among the boarded-out only 2 per cent. recovered.

### A Great New Hospital for Vienna.

On Tuesday the Emperor Francis Joseph laid the foundation-stone of the new General and University Hospital at Vienna, which in process of time is to take the place of the Königliche und Kaiserliche Allgemeine Krankenhaus in the Alserstrasse, built 120 years ago. His Majesty, who, like his father, has always manifested a keen personal interest in the hospitals in his country, has thus associated himself with a movement of the greatest importance in the Austrian capital. The old hospital owes its existence as a hospital to the munificence of the Emperor Joseph, who adapted it from its original purpose as a workhouse and converted it into a hospital, asylum, and foundling institution. In 1834 all those departments not strictly appertaining to the sick were removed to other management. The hospital, which is under State control, now contains 2,000 beds. The new building, which is expected to cost close upon two million pounds, and to take 10 years to erect, is to consist of 40 pavilions, including 18 clinical institutes, with the best possible arrangements for teaching purposes and demonstration. In each operating amphitheatre there will be room for 250 students. The patients in the clinical institutes for infectious diseases will be separated from both professor and students by a glass screen. Every clinical institute will have a large ambulatorium, and there will also be in each flat roofs, where patients may lie in the open air whenever weather and temperature allow it. The area at the disposal of the authorities is nearly 60 acres, or more than twice the area of the present Krankenhaus, but it appears that there will be fewer beds. A striking feature will be the gardens, which will cover 48 acres. In this respect, at all events, the Vienna General Hospital will possess advantages which could not very well be enjoyed by institutions in England adjacent to populous cities. Austrian physicians and surgeons are hoping that, when the new hospital is completed and equipped, Vienna may gain in reputation as one of the chief medical centres of the world.

### Asepsis and the Oath.

The obvious objection to the kissing of a book which has, in many cases for long years, received similar salutations from hundreds or thousands of other people of various shades of uncleanliness has at various times stimulated suggestions as to how this insanitary proceeding can be avoided. Occasionally a witness of fresh and fertile mind has carried to the Court his own private copy of the New Testament, and has insisted on using this in subscribing to the judicial demand. Apart from other inconveniences, the process leads to delay, as the Court, before allowing this proceeding, must be satisfied that the volume offered is really what it pretends to be. It is only a few months ago that a young lady witness professed her desire to take the oath by kissing a book she had brought with her, and this on examination proved to be a work of private authorship. However, in accordance with precedent, she had her own way, at least in some measure, for whilst the Court insisted upon the use of the orthodox, if grimy, volume, she followed up her concession in this direction by a chaste salute applied to the pages of her private manual. More recently a scientific enthusiast has suggested that the official copy of the New Testament should be so bound as to permit of it being washed in some antiseptic solution, presumably after each attesting osculation. Whether the judicial authorities will permit the introduction into the law courts of the apparatus and ritual of modern surgery may well be doubted. All these suggestions and devices are altogether unnecessary if witnesses would only remember that it is entirely at their option to decline to "kiss the book" and to demand to be sworn with uplifted hand. It is particularly surprising that police surgeons do not avail themselves of this arrangement. Their duties often carry them into the witness-box, and they have an intimate knowledge of the personal habits of many who find themselves in the same situation. If some enterprising spirit among them would only lead a crusade against the present practice and would convert his colleagues, he would doubtless have the satisfaction of seeing his example followed by the general public. The reform would be in the direction of wise sanitation and common-sense. A sanitary oath can be obtained without antiseptic washings and sterilised Bibles.

